# Cytotoxicity of Snake Venoms and Cytotoxins From Two Southeast Asian Cobras (*Naja sumatrana*, *Naja kaouthia*): Exploration of Anticancer Potential, Selectivity, and Cell Death Mechanism

**DOI:** 10.3389/fmolb.2020.583587

**Published:** 2020-11-11

**Authors:** Ho Phin Chong, Kae Yi Tan, Choo Hock Tan

**Affiliations:** ^1^Venom Research and Toxicology Lab, Department of Pharmacology, Faculty of Medicine, University of Malaya, Kuala Lumpur, Malaysia; ^2^Protein and Interactomics Lab, Department of Molecular Medicine, Faculty of Medicine, University of Malaya, Kuala Lumpur, Malaysia

**Keywords:** equatorial spitting cobra, monocled cobra, cytotoxin purification, cardiotoxin, flow cytometry

## Abstract

Venoms of cobras (*Naja* spp.) contain high abundances of cytotoxins, which contribute to tissue necrosis in cobra envenomation. The tissue-necrotizing activity of cobra cytotoxins, nevertheless, indicates anticancer potentials. This study set to explore the anticancer properties of the venoms and cytotoxins from *Naja sumatrana* (equatorial spitting cobra) and *Naja kaouthia* (monocled cobra), two highly venomous species in Southeast Asia. The cytotoxicity, selectivity, and cell death mechanisms of their venoms and cytotoxins (NS-CTX from *N. sumatrana*: NS-CTX; *N. kaouthia*: NK-CTX) were elucidated in human lung (A549), prostate (PC-3), and breast (MCF-7) cancer cell lines. Cytotoxins were purified through a sequential fractionation approach using cation-exchange chromatography, followed by C_18_ reverse-phase high-performance liquid chromatography (HPLC) to homogeneity validated with sodium dodecyl sulfate-polyacrylamide gel electrophoresis, and identified by liquid chromatography-tandem mass spectrometry (LCMS/MS). The cobra venoms and their respective cytotoxins exhibited concentration-dependent growth inhibitory effects in all cell lines tested, with the cytotoxins being more potent compared to the corresponding whole venoms. NS-CTX and NK-CTX are, respectively, P-type and S-type isoforms of cytotoxin, based on the amino acid sequences as per LCMS/MS analysis. Both cytotoxins exhibited differential cytotoxic effects in the cell lines tested, with NS-CTX (P-type cytotoxin) being significantly more potent in inhibiting the growth of the cancer cells. Both cytotoxins demonstrated promising selectivity only for the A549 lung cancer cell line (selectivity index = 2.17 and 2.26, respectively) but not in prostate (PC-3) and breast (MCF-7) cancer cell lines (selectivity index < 1). Flow cytometry revealed that the A549 lung cancer cells treated with NS-CTX and NK-CTX underwent necrosis predominantly. Meanwhile, the cytotoxins induced mainly caspase-independent late apoptosis in the prostate (PC-3) and breast (MCF-7) cancer cells lines but lacked selectivity. The findings revealed the limitations and challenges that could be faced during the development of new cancer therapy from cobra cytotoxins, notwithstanding their potent anticancer effects. Further studies should aim to overcome these impediments to unleash the anticancer potentials of the cytotoxins.

## Introduction

Cancer is a disease characterized by abnormal and uncontrollable cell division. It has become the second leading cause of death globally, with 9.6 million deaths reported by the WHO in 2018 (Bray et al., 2018). Conventional cancer treatments, such as radiotherapy and chemotherapy, are unavoidably accompanied with adverse effects as they are designed for mass killings of cancer cells while lacking specificity. To further complicate matters, cancer cells may acquire resistance to chemotherapeutic treatments. Thus, the pursuit for a cancer-specific, non-resistant, and potent compound has been the cornerstone in anticancer drug discovery (Liberio et al., 2013). In this context, snake venom proteins are promising candidates for novel anticancer agents (Li et al., 2018). Snake venoms consist of evolutionary fine-tuned, pharmacologically active proteins and peptides to most mammalian cells (Mackessy, 2009; Casewell et al., 2013). The mammalian system-targeting property of snake venom proteins is typically specific and selective, thus making them a repertoire of bioactive molecules for the discovery of new therapeutic agents (Lewis and Garcia, 2003).

In Asia and Africa, envenomation by cobras (*Naja* spp.) causes fatal paralysis and debilitating complications resulted from extensive tissue necrosis (Ranawaka et al., 2013; World Health Organization [WHO], 2016). The tissue-necrotizing effect observed in cobra envenomation suggests cobra venoms contain cytotoxic components that can be further explored for anticancer potential. Studies have shown that virtually all cobra species produce a significant amount of cytotoxins in their venom proteomes, with abundances ranging from 20% to as high as ∼80% of the total venom proteins (Petras et al., 2011; Huang et al., 2015; Dutta et al., 2017; Tan et al., 2019; Tan et al., 2020). Cobra venom cytotoxins are polypeptides of 60–70 amino acid residues held by four disulfide crosslinks, forming a three-protruding fingers-like structure from the core of the molecule and is grouped under the three-finger toxin superfamily (Antil-Delbeke et al., 2000; Servent et al., 2000). Its unique structure gives rise to its diverse pharmacological actions, including cytolytic activity through pore formation on cell membranes, and disruption of membrane lipid bilayer integrity, thus initiating cell death (Konshina et al., 2011; Chan et al., 2016). Its cytotoxic properties, nevertheless, imply the possibility to harness cobra cytotoxins in the development of new anticancer drugs.

Earlier studies had shown that cytotoxins from a few cobra species, i.e., *Naja atra* (Chen et al., 2009; Tsai et al., 2016), *Naja haje* (Feofanov et al., 2005), *Naja naja* (Das et al., 2013), and *Naja oxiana* (Ebrahim et al., 2016) were cytotoxic to some cancer cell lines. The anticancer properties of various cytotoxin subtypes from most other cobra species, however, remain largely uncharacterized. In this regard, the two most common cobra species in Southeast Asia, i.e., *Naja sumatrana* (equatorial spitting cobra) and *Naja kaouthia* (monocled cobra), which cause significant tissue necrosis in envenomed patients (MOH Malaysia, 2017), are valuable sources of venoms to elucidate the prospect of cytotoxin-derived anticancer therapeutics (Ebrahim et al., 2016). This study set to investigate the anticancer properties of the venoms of *N. sumatrana* and *N. kaouthia* as well as their purified cytotoxins in the breast, prostate, and lung cancer cell lines. The study further delved into the specificity and selectivity of the cytotoxins and verified the cell death mechanisms underlying their cytotoxic activities.

## Materials and Methods

### Snake Venom

*Naja kaouthia* (NK) venom was supplied by Queen Saovabha Memorial Institute (Bangkok), an antivenom manufacturing facility in Thailand. *N. sumatrana* (NS) venom was sourced from specimens in Negeri Sembilan (south-western region of Peninsular Malaya) in Malaysia, provided by local snake keepers. The venoms were both pooled samples of the respective species comprising multiple adult snakes of mixed sexes. The collection of venom was conducted in accordance with protocol approved by the Institutional Animal Use and Care Committee (IACUC) of Faculty of Medicine, University of Malaya, Malaysia (code: #2013-11-12/PHAR/R/TCH). Both venoms were lyophilized samples stored at −20°C until use.

### Fractionation of Cobra Venoms With Resource S Cation-Exchange Chromatography

*Naja sumatrana* and *N. kaouthia* venoms were separately reconstituted in eluent A [20 mM 2-(N-morpholino) ethanesulfonic acid, pH 6] and fractionated with Resource S cation-exchange chromatography (GE Healthcare, Sweden) applying the Shimadzu LC-20AD high-performance liquid chromatography (HPLC) system (Kyoto, Japan). The system was pre-equilibrated with eluent A, followed by elution with eluent B (0.8 M sodium chloride in 20 mM MES, pH 6) using a linear-gradient flow of 0–30% B from 5 to 40 min and 30–100% from 40 to 65 min. The flow rate was set at 1 ml/min. Protein elution was monitored at 280 nm absorbance, and the fractions containing cytotoxins (CTX) were manually collected with reference to the venom proteomes reported earlier for *N. sumatrana* (Yap et al., 2014) and *N. kaouthia* (Tan et al., 2016b). The protein fractions were diafiltered with Vivaspin (Sartorius, Göttingen, Germany), freeze-dried, and subjected to further purification and protein identification.

### Further Purification of Cytotoxins With C_18_ Reverse-Phase HPLC

Cytotoxin-containing fractions eluted from Resource S cation-exchange chromatography (Fraction 8 for *N. sumatrana* and *N. kaouthia* venoms) were reconstituted in Solvent A [0.1% trifluoroacetic acid (TFA) in ultrapure water]. The cytotoxins were subjected to further purification using LiChrospher WP 300 C_18_ (5 μm pore size) reverse-phase column applying the Shimadzu LC-20AD HPLC system (Kyoto, Japan). The column was pre-equilibrated with solvent A, followed by elution with solvent B (0.1% TFA in acetonitrile), with a linear gradient of 5% for 10 min, 5–15% for 20 min, 15–45% for 120 min, and 45–70% for 20 min, as described by Tan C.H. et al. (2017). The elution rate was set at 1 ml/min. Protein elutions were monitored at 215 nm and the protein fraction containing cytotoxins was manually collected, lyophilized, and stored at −20°C until further use.

### Validation of Cytotoxins With Sodium Dodecyl Sulfate-Polyacrylamide Gel Electrophoresis (SDS-PAGE) and Liquid Chromatography-Tandem Mass Spectrometry (LCMS/MS)

Five micrograms (μg) of the purified NS-CTX and NK-CTX were subjected to 15% sodium dodecyl sulfate-polyacrylamide gel electrophoresis (SDS-PAGE) under reducing conditions for purity check. The molecular weights of the proteins were calibrated with the ExcelBand^TM^3-color Broad Range Protein Marker (5–245 kDa, SMOBIO, Taiwan). Electrophoresis was conducted at 90 V for 2.5 h. Visualization of proteins was achieved by Coomassie Brilliant Blue R-250 stain.

Separately, 5 μg of the purified NS-CTX and NK-CTX were subjected to LCMS/MS for protein identification. The CTXs were reduced with dithiothreitol (DTT), alkylated with iodoacetamide (IA), and digested with MS grade Pierce trypsin protease (Thermo Scientific Pierce, Rockford, IL, United States) according to the manufacturer’s protocol. The digested peptides were then desalted and extracted with Millipore ZipTip^®^ C_18_ pipette tip (Merck, United States). Digested peptides were subsequently subjected to nano-electrospray ionization-liquid chromatography-tandem mass spectrometry (nano-ESI-LC-MS/MS) with Agilent 1200 HPLC-Chip/MS Interface, coupled with Agilent 6520 Accurate-Mass Q-TOF LC/MS system, as previously described by Tan C.H. et al. (2017). The sample was loaded into a 300 Å, C_18_ enrichment column, followed by 75 μm × 150 mm analytical column (Agilent No. G4240-62010); 0.1% formic acid in water and 90% acetonitrile in water with 0.1% formic acid were used as eluting solvents A and B, respectively. A stepwise linear gradient was applied: 3–50% solvent B for 30 min, 50–95% for 2 min, and 95% for 5 min. The ion polarity was set to positive. Drying gas was set to 325°C with a drying gas flow rate of 5 l/min. Capillary voltage was adjusted to 1995 V while the fragmentor voltage was set to 175 V. Spectra acquisition was done in MS/MS mode with MS scan range of 110–3000 and 50–3000 *m/z*. Precursor change selection was set to include a doubly, triply, or more charged state, excluding the precursors 922.0098 *m/z* (*z* = 1) and 121.0509 (*z* = 1) which were used as reference ions. Data were obtained with MH^+^ range between 600 and 4000 Da and processed using Agilent Spectrum Mill MS Proteomics Workbench software packages. The peptide finger mapping was searched against the non-redundant NCBI database of Serpentes (taxid: 8570)^1^. Carbamidomethylation of cysteine was set as a single modification. Protein identification was validated with the following filtering parameters: protein score > 11, peptide score > 6, and scored peak intensity (SPI) > 60%. The peptide information of cytotoxin identification was included as Supplementary File S3.

The obtained tryptic peptides were further aligned with most similar toxin sequences retrieved from UniProtKB depository^2^ using Jalview (Waterhouse et al., 2009) and MUSCLE (Edgar, 2004) software (version 2.10.5). The categorization of cytotoxins as P-type or S-type was determined based on the presence of Proline^30^ and Serine^28^, respectively (Dubovskii et al., 2005).

### Cell Culture

Both cancer and normal cell lines were obtained from ATCC (Manassas, VA, United States). Breast cancer epithelial cells (MCF-7, ATCC^®^ HTB-22^TM^) were cultured in DMEM (Nacalai tesque, Kyoto, Japan) with 10% FBS (TICO Europe, Amstelveen, Netherlands) and 1% penicillin-streptomycin (Nacalai tesque; Kyoto, Japan); lung (A549, ATCC^®^ CCL-185^TM^) and prostate (PC-3, ATCC^®^ CCL-1435^TM^) cancer epithelial cells were maintained in RPMI (Nacalai Tesque, Kyoto, Japan) with 10% FBS and 1% penicillin-streptomycin; normal breast epithelial cells (184B5, ATCC^®^ CRL-8799^TM^) were grown in MEBM^TM^ (Lonza, Basel, Switzerland) with 10% FBS and 1% penicillin-streptomycin; normal lung epithelial cells (NL20, ATCC^®^ CRL-2503^TM^) raised in Ham’s F-12 medium (Nacalai tesque, Kyoto, Japan) with 10% FBS and 1% penicillin-streptomycin. Normal prostate epithelial cells (RWPE-1, ATCC^®^ CRL-11609^TM^) were cultivated in Gibco^TM^ Keratinocyte-SFM (Thermo Fisher, Waltham, MA, United States) with 1% penicillin-streptomycin. All cells were grown in sterile flasks (T25/T75) at <90% confluence at 37°C with 5% CO_2_ in a humidified atmosphere. Cells were passaged in accordance with ATCC guidelines, with trypsin-EDTA (0.05% trypsin and 0.02% EDTA; Nacalai tesque, Kyoto, Japan) and cryopreserved in its respective complete medium with 10% DMSO (Friendemann Schmidt, WA, United States) in Mr. Frosty^TM^ freezing container (Nalgene^®^ Thermo Scientific Pierce, Rockford, IL, United States) at −80°C.

### Cytotoxicity on Normal and Cancer Cell Lines

Cytotoxicity was tested with MTT [3-(4,5-dimethylthiazol-2-yl)-2,5-diphenyltetrazolium bromide; Merck Millipore, MA, United States] assay. Twenty-four hours prior to venom treatment, 100 μl aliquots of optimum cells (3000–4000 cells/well) were seeded into 96-well microtiter plates. Cells were introduced with 200 μl of diluted suspensions of crude venom or purified CTX of *N. sumatrana* (NS-CTX) and *N. kaouthia* (NK-CTX) in complete culture medium (μg/ml) or equal volume of complete medium without venom as the negative control or 5-fluorouracil (5FU; Sigma–Aldrich, St. Louis, MO, United States) as positive control. Cytotoxicity was assessed 72 h post-treatment with 10% MTT, incubated at 37°C in the dark for 3 h. Cells were ruptured and the purple formazan was dissolved with 200 μl of DMSO. Finally, data were quantified by obtaining the absorbance at 570 nm using Chameleon^TM^ VS Multilabel microplate reader (Hidex, Turku, Finland). All assays were performed in triplicates. Cell viability percentage was calculated as:

$$\frac{\left(A\,b\,s\,o\,r\,b\,a\,n\,c\,e\,o\,f\,e\,x\,p\,e\,r\,i\,m\,e\,n\,t\,a\,l\,s\,a\,m\,p\,l\,e\right)-\left(A\,b\,s\,o\,r\,b\,a\,n\,c\,e\,o\,f\,b\,l\,a\,n\,k\,s\,a\,m\,p\,l\,e\right)}{\left(A\,b\,s\,o\,r\,b\,a\,n\,c\,e\,o\,f\,u\,n\,t\,r\,e\,a\,t\,e\,d\,s\,a\,m\,p\,l\,e\right)-\left(A\,b\,s\,o\,r\,b\,a\,n\,c\,e\,o\,f\,b\,l\,a\,n\,k\,s\,a\,m\,p\,l\,e\right)}\times \,100\%$$

Half maximal inhibitory concentration (IC_50_) and statistical significance was determined using one-way ANOVA, with Bonferroni post-test (95% confidence interval, *p* < 0.05 for significance) via GraphPad Prism (Version 5.03). The results were expressed as mean of triplicates ± SEM.

### Phosphatidylserine Exposure

Flow cytometry with Annexin-V-fluorescein isothiocyanate (FITC) conjugated/propidium iodide stains was used to determine phosphatidylserine externalization. The cells were grouped as viable (annexin V^–^/PI^–^), early apoptosis (annexin V^+^/PI^–^), late apoptosis/secondary necrosis (annexin V^+^/PI^+^), or necrosis (annexin V^–^/PI^+^). In brief, 500,000 cancer cells (MCF-7, A549, and PC-3) were seeded in sterile T25 flasks 24 h prior to treatment with their respective IC_50_ of purified cytotoxins or 5FU (positive control) for 72 h. The cells were then washed twice with cold Dulbecco’s Phosphate Buffered Saline (PBS; Cytonex^TM^, Malaysia), resuspended in binding buffer, and stained for 15 min in the dark with Annexin V and propidium iodide as per manufacturer’s instructions (FITC Annexin V Apoptosis Detection Kit I; BD Bioscience, NJ, United States); 10,000–15,000 events were compensated and acquired with flow cytometer (FACSCanto^TM^ II, BD Bioscience, NJ, United States), equipped with blue laser (488 nm) and emission filters of 488/10 for FITC and 585/42 (556 LP) for PI. The FACSDiva software (Version 6.1.3; BD bioscience, NJ, United States) was utilized in data analysis.

### Data Availability

The mass spectrometry proteomics data have been deposited to the ProteomeXchange Consortium^3^ via the iProX partner repository (Ma et al., 2018) with the dataset identifier PXD021350.

## Results

### Cytotoxicity of *Naja sumatrana* and *Naja kaouthia* Venom in Cancer Cell Lines

The venoms of *N. sumatrana* (NS) and *N. kaouthia* (NK) exhibited concentration-dependent cytotoxic activities toward the three cancer cell lines tested, with varying half maximal inhibitory concentrations (IC_50_) (Supplementary File S1). Both cobra venoms were most potent in inhibiting the growth of lung cancer cell line (A549, IC_50_ = 2.21-3.26 μg/ml), moderately cytotoxic to the prostate cancer cell line (PC-3, IC_50_ = 5.58-9.88 μg/ml), and least cytotoxic toward breast cancer cell line (MCF-7, IC_50_ = 10-34.47 μg/ml) (Supplementary File S2). N. sumatrana venom showed a wide range of cytotoxicity across the three cell lines, where the growths of A549 and PC-3 were significantly inhibited 6–15 times more potently than MCF-7 (A549: IC_50_ = 2.21 ± 0.25 μg/ml; PC-3: IC_50_ = 5.58 ± 0.67 μg/ml; MCF-7: IC_50_ = 34.47 ± 1.59 μg/ml) (*p* < 0.001). N. kaouthia venom had a narrower range of cytotoxicity across the three cancer cell lines, with the activity being highest in the A549 cell line (IC_50_ = 3.26 ± 0.39 μg/ml) (*p* < 0.001).

### Purification and Validation of Cytotoxins

**FIGURE 1:**
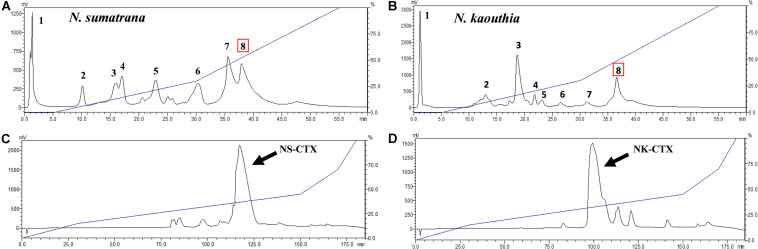
Purification of cytotoxins from *Naja sumatrana* and *Naja kaouthia* venoms with sequential high-performance liquid chromatography (HPLC). **(A,B)** Resource S cation-exchange HPLC of N. sumatrana and N. kaouthia venoms, respectively. **(C,D)** C_18_ reverse phase-HPLC of CTX-containing Fraction 8 eluted from cationic-exchange chromatography of *N. sumatrana* and *N. kaouthia* venoms, respectively. Arrows indicate the major basic cytotoxins purified.

N. sumatrana and N. kaouthia venoms were both resolved by cation-exchange chromatography into eight fractions (Figures 1A,B). Cytotoxins in Fraction 8 of the respective venoms were further purified with C_18_ reverse-phase HPLC. The major CTXs of *N. sumatrana* (NS-CTX) and *N. kaouthia* (NK-CTX) were eluted within 110–125 min (Figure 1C) and 90–115 min (Figure 1D), respectively.

**FIGURE 2:**
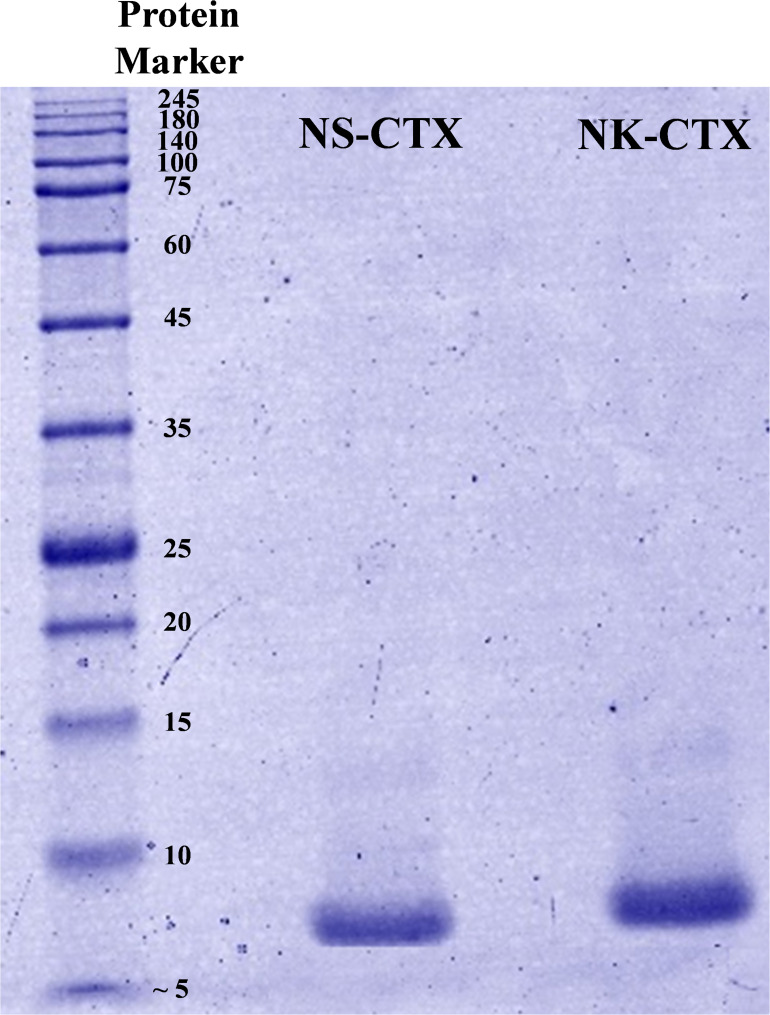
Sodium dodecyl sulfate-polyacrylamide gel electrophoresis of cytotoxins purified from *Naja sumatrana* (NS-CTX) and *Naja kaouthia* (NK-CTX) venoms under reducing condition.

**FIGURE 3:**
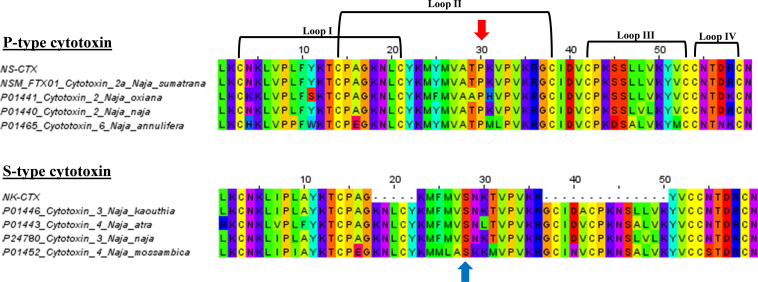
Multiple sequence alignments of NS-CTX and NK-CTX, visualizing P-type and S-type cytotoxins. Arrow in red indicates the Proline^30^ of P-type cytotoxin; blue arrow indicates the Serine^28^ of S-type cytotoxin.

In SDS-PAGE, both purified NS-CTX and NK-CTX showed a homogenous protein band of approximately 7 kDa (Figure 2). With nano-ESI LCMS/MS, the identities of NS-CTX and NK-CTX were validated as cobra cytotoxin (Table 1). The amino acid sequences of NS-CTX and NK-CTX exhibited high homology to the venom-gland transcriptome of *N. sumatrana*, NSM_FTX01, and P01446 (*N. kaouthia*), respectively (Table 1). Multiple sequence alignment showed the presence of a Pro^30^ residue at the second loop of NS-CTX and a Ser^28^ residue in NK-CTX, consistent with the categorization of P-type and an S-type cytotoxin, respectively (Figure 3).

**TABLE 1:** Identification of cytotoxins from *Naja sumatrana* (NS-CTX) and *Naja kaouthia* (NK-CTX) by nano-ESI-LCMS/MS.

**Protein designated identity**	**Protein score**	**Protein name matched**	**Accession code (species)**	**Matched distinct peptides**	**Coverage (%)**
**NS-CTX**	166.03	Cytotoxin 1	NSM_FTX01^a^	LKCNKLVPLFYK	100.00
			(*N. sumatrana*)	CNKLVPLFYK	
				LVPLFYKTCPAGKNLCYK	
				LVPLFYK	
				MFMVATPKVPVKR	
				MFMVATPKVPVK	
				MFMVATPK	
				GCIDVCPKSSLLVK	
				SSLLVKYVCCNTDRCN	
				SSLLVK	
				YVCCNTDRCN	
				YVCCNTDR	
**NK-CTX**	120.68	Cytotoxin 3	P01446	LKCNKLIPLAYK	66.6
			(*N. kaouthia*)	CNKLIPLAYK	
				LVPLFYK	
				LIPLAYKTCPAGK	
				LIPLAYK	
				MFMVSNKTVPVKR	
				YVCCNTDRCN	
				YVCCNTDR	

*^*a*^Accession code retrieved from *N. sumatrana* venom-gland transcriptome (Chong et al., 2019). LCMS search data are included as Supplementary File S3.*

### Cytotoxicity of Cytotoxins From *Naja sumatrana* and *Naja kaouthia*

**FIGURE 4:**
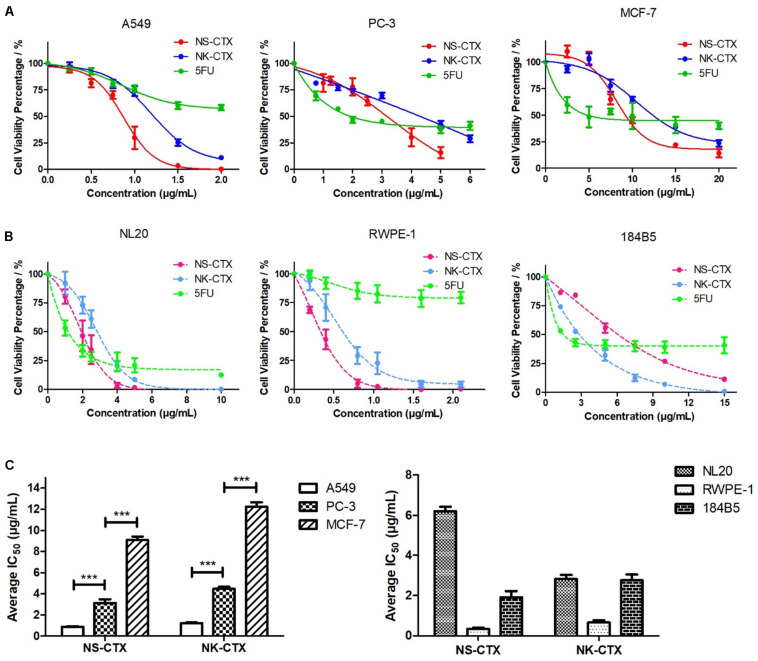
Cytotoxicity of NS-CTX, NK-CTX, and 5-fluorouracil (5FU) on cancer and normal cell lines. **(A)** Cell viability plot for cancer cell lines (A549, PC-3, and MCF-7). **(B)** Cell viability plot for normal cell lines (NL20, RWPE-1, and 184B5). **(C)** Half maximal inhibitory concentrations (IC_50_) of cell lines treated with NS-CTX and NK-CTX. All assays involved three technical and biological triplicates. One-way ANOVA with Bonferroni *post hoc* test was used to determine statistical significance across three cancer cell lines (*** indicates *p* < 0.001).

NS-CTX, NK-CTX, and 5FU (positive control) showed dose-dependent cytotoxic activities in the cancer cell lines tested (A549, PC-3, and MCF-7) and their corresponding normal cell lines (breast: 184B5; lung, NL20; prostate, RWPE-1) (Figure 4). In cancer cell lines, both NS-CTX and NK-CTX demonstrated increasing cytotoxicity in the following order: MCF-7 (IC_50_ = 9.10–12.23 μg/ml) < PC-3 (IC_50_ = 3.13–4.46 μg/ml) < A549 (IC_50_ = 0.88–1.22 μg/ml) (Table 2). NS-CTX was generally more potent than NK-CTX in cytotoxicity. The normal cell lines of breast (184B5: IC_50_ = 2.83–6.21 μg/ml) and prostate (RWPE-1: IC_50_ = 0.35–0.65 μg/ml) were more susceptible to NS-CTX and NK-CTX compared to their respective cancer cell lines, demonstrating no cancer cell-type selectivity. In contrast, NS-CTX and NK-CTX showed selectivity for lung cancer cell line (A549) over the normal lung cell line (NL20), with a selective index of 2.17 and 2.26, respectively (Table 2).

**TABLE 2:** Half maximal inhibitory concentrations (IC_50_) and selectivity index of NS-CTX and NK-CTX in cancer and normal lung, prostate, and breast cell lines.

**Tissue origin**	**Cell line**	**NS-CTX**		**NK-CTX**	
		**IC_50_ (μ g/ml)**	**Selectivity index**	**IC_50_ (μ g/ml)**	**Selectivity index**
Lung	A549	0.88 ± 0.06	2.17	1.220.09	2.26
	NL20	1.91 ± 0.52		2.760.49	
Prostate	PC-3	3.13 ± 0.58	0.11	4.460.36	0.15
	RWPE-1	0.35 ± 0.08		0.650.20	
Breast	MCF-7	9.10 ± 0.56	0.68	12.230.76	0.23
	184B5	6.21 ± 0.37		2.830.34	

*Values were expressed in mean ± SEM.*

**FIGURE 5:**
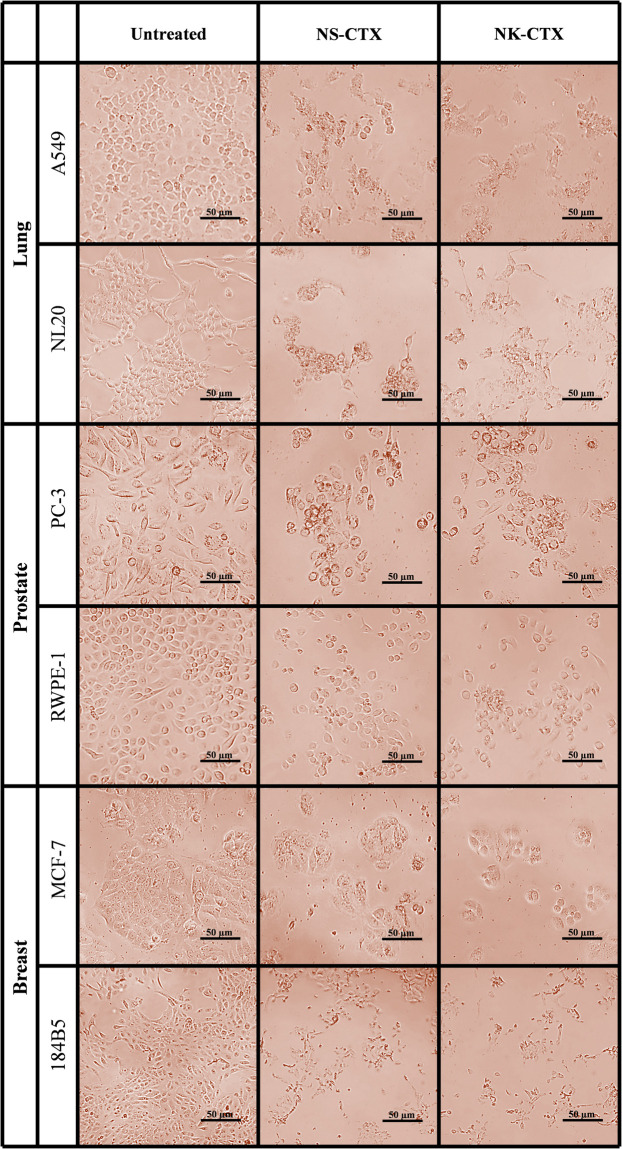
Microscopic appearance of cancer and normal cell lines of lung, prostate, and breast treated with NS-CTX and NK-CTX at their respective median inhibitory concentrations (IC_50_).

Microscopic observation revealed morphological changes of cell clustering and shriveling in both cancer and normal lung (A549 and NL20) and prostate (PC-3 and RWPE-1) cell lines treated with NS-CTX and NK-CTX (Figure 5). In breast cell lines (MCF-7 and 184B5), a small degree of cell aggregation was observed when treated with NS-CTX and NK-CTX.

### Flow Cytometry Analysis of Cancer Cells Upon Cytotoxin Treatment

**FIGURE 6:**
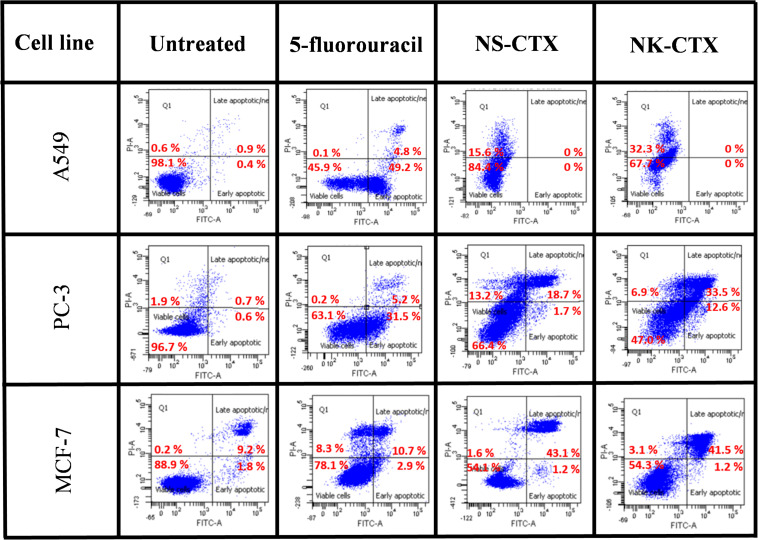
Dot plots showing the flow cytometric cell death mechanism of cancer cell lines (A549, PC-3, and MCF-7) induced by NS-CTX and NK-CTX treatment. All cell lines were treated with IC_50_ of NS-CTX and NK-CTX for 72 h. Cells were stained with Annexin-V and/or PI. Non-treated cells were used as negative control, and cells treated with 5-fluorouracil were used as positive control for apoptosis.

Flow cytometry demonstrated varying cell death mechanisms, predominantly necrosis and late apoptosis in the cancer cell lines upon treatment with NS-CTX and NK-CTX (Figure 6). Both NS-CTX and NK-CTX induced primarily necrosis in the A549 cell line, with a twofold higher potency observed in the latter. In the PC-3 cell line, NS-CTX induced comparable levels of late apoptosis and necrosis, while more prominent late apoptosis was noted in NK-CTX-treated cells. Both NS-CTX and NK-CTX induced mainly late apoptosis in the MCF-7 cell line. In parallel, caspase 3/7 activities were not detected in the cell lines upon treatment with NS-CTX and NK-CTX (data not shown).

## Discussion

The present study demonstrated the cytotoxic activities of *N. sumatrana* and *N. kaouthia* venoms in lung (A549), prostate (PC-3), and breast cancer (MCF-7) cell lines. The venom cytotoxic effects were more prominent in A549 and PC-3 cell lines than MCF-7 cell line, suggesting higher tissue specificity toward the lung and prostate cancer cell lines. The major cytotoxins from both venoms were purified to homogeneity, validated by LCMS/MS, and investigated for their cytotoxic properties in the cancer cell lines. The respective cytotoxins also exhibited a similar trend of tissue specificity as with their venoms toward the A549 and PC-3 cancer cell lines, unveiling the therapeutic potentials of cobra-derived cytotoxins against these cancers. The purified CTXs were apparently more potent (with lower IC_50_) compared to the respective crude venoms, supporting that these proteins are the main cytotoxic component in the cobra venoms implicated in tissue necrosis (Tan and Tan, 2016). Sequences of the two cytotoxins indicated that the NS-CTX and NK-CTX belong to P-type and S-type subgroups of CTX, respectively. The P-type and S-type cytotoxins vary in their amino acid sequence at the tip of loop II: the former is characterized by the presence of Pro^31^ within the phospholipid binding site near the tip of loop II, whereas the latter has Ser^29^ within the same but more hydrophilic region. The binding of virtually all CTX to phospholipid membranes involves a phospholipid binding site at loop I; however, the presence of Pro^31^ in P-type CTX gave rise to an additional phospholipid binding site at loop II, thus enabling the P-type CTX to exhibit higher fusion and binding activity than the S-type CTX to cell membrane (Chien et al., 1994; Suzuki-Matsubara et al., 2016). In this study, the higher potency of NS-CTX compared with NK-CTX is therefore consistent with the differential cytotoxicity reported previously for the P-type and S-type of CTX. P-type cytotoxins such as NS-CTX presumably exert a deeper penetration into the lipid bilayer of anionic phospholipid-containing membrane and destabilize the membrane more efficiently (Chien et al., 1994; Almeida et al., 2002; Dubovskii et al., 2005).

Recent proteomic studies validated that CTXs are indeed abundant in *N. sumatrana* and *N. kaouthia* venoms of various locales, constituting at least 20% of the total venom proteins (Yap et al., 2014; Tan et al., 2016a; Liu et al., 2017; Xu et al., 2017). The two major subgroups of CTX, i.e., P-type and S-type CTX, distribute variably among different *Naja* species. In this study, the purification of the P-type CTX and S-type CTX from *N. sumatrana* and *N. kaouthia* venoms, respectively, is consistent with the venom proteomes (Yap et al., 2014; Tan et al., 2015) and venom-gland transcriptomes of the respective species (Tan K.Y. et al., 2017; Chong et al., 2019). The genome of the Indian cobra (*N. naja*), on the other hand, indicated that the genes of both P-type and S-type CTX could well be conserved in other cobra species (Suryamohan et al., 2020). Nevertheless, environmental factors, particularly diet, can impact protein expression and modulate the dominant phenotypic expression of CTX in the cobra venom, contributing to the differential cytotoxicity of the venom as observed in this study.

The discovery of toxin compounds with low IC_50_ comparable to or superior than commercially available anticancer drugs is not uncommon, and this has piqued great interest into the medicinal attribute of toxins as anticancer therapeutics (Ebrahim et al., 2016; Attarde and Pandit, 2017). The potent cytotoxicity of toxins, however, may cause collateral damage to normal or non-cancerous cells, defying the therapeutic use of these new drug candidates. Selectivity study is hence warranted, and in this study, both NS-CTX and NK-CTX exhibited significantly higher IC_50_ values in the normal lung NL20 cell line compared with the cancerous A549 cell line, indicating good selective cytotoxicity for lung cancer cell line. The selectivity of cytotoxins, however, was absent in the prostate (PC-3) and breast (MCF-7) cancer cell lines, implying limited application of the toxins against these cancer cells. The higher selective cytotoxicity of the cytotoxins in A549 cell lines could be attributed to the unique characteristic of non-small lung cancer cell membrane, in which phosphatidylinositols are present abundantly (Marien et al., 2015). Earlier, Wang et al. (2006) reported that cobra cytotoxin interacted and bound preferentially with negatively charged lipids like phosphatidylinositol. It is possible that the preferred binding facilitated the membrane insertion and internalization of CTX into the A549 cancer cell lines.

In flow cytometry, both NS-CTX and NK-CTX induced late apoptosis in PC-3 and MCF-7. The finding is consistent with a cytotoxin isolated from *N. naja* (Das et al., 2013), *N. atra* (Yang et al., 2006), and *N. oxiana* (Ebrahim et al., 2014), which demonstrated late apoptosis as the primary cell death mechanism in CTX-treated leukemia and breast cancer cell lines. However, the late apoptosis was not observed along with the upregulation of caspase 3/7 activity in the present study. This finding indicated that the late apoptosis in PC-3 and MCF-7 by NS-CTX and NK-CTX is likely mediated through caspase-independent pathways, possibly through AIF, endonuclease G, or HtrA2 (Lorenzo and Susin, 2004; Chen and Wong, 2009; Kim et al., 2009). The elucidation of the exact pathways requires further investigations. Although NS-CTX and NK-CTX showed commendable cell-type selectively toward the lung A549 cell line, the flow cytometric finding revealed that the cells predominantly underwent necrosis, consistent with the absence of phosphatidylserine exposure and caspase activation.

To further understand the anticancer properties and cell death mechanisms of cobra venoms and their cytotoxins, we reviewed and tabulated findings from related studies involving a variety of *Naja* spp. reported thus far, which included *N. atra*, *N. haje*, *N. naja*, *Naja nigricollis*, *N. oxiana*, and *N. sumatrana* as well as *N. kaouthia* (current study), tested on assorted cancer cell lines (Table 3). Presently, most studies on the cytotoxicity and the implicated cell-death mechanisms were reported for *N. atra* cytotoxins (Tsai et al., 2006; Chen et al., 2009; Chien et al., 2009; Chiu et al., 2009; Lin et al., 2010; Wu et al., 2013; Liu et al., 2019) and *N. naja* (Das et al., 2013), while studies on other *Naja* spp. cytotoxins were largely limited to cytotoxicity IC_50_ reporting in cancer cells (Feofanov et al., 2005; Ebrahim et al., 2016; Attarde and Pandit, 2017; Conlon et al., 2020). The present study is the first to elucidate the cytotoxicity, selectivity, and cell death mechanism of the purified cytotoxins of *N. sumatrana* and *N. kaouthia*, tested in lung (A549), prostate (PC-3), and breast (MCF-7) cancer cell lines. Overview of the IC_50_ of cytotoxins from various *Naja* spp. showed wide-ranging differential cytotoxicity (IC_50_ values between 0.88 and 12.23 μg/ml) in the different mammalian cell lines, treated with cytotoxin between 12 and 72 h. However, a much broader range of IC_50_ was seen in the purified cytotoxins of *N. oxiana*, *N. haje*, and *N. nigricollis*, with values wide ranging from 0.33 to 132 μM tested in lung, peripheral blood, breast, and colon cancer cell lines (Feofanov et al., 2005; Conlon et al., 2020). The cytotoxic potentials and cell death pathways were, apparently, highly variable depending on the types of cell, concentrations of cytotoxin, durations of treatment, and most likely, species of the cobra too. Although all cobra cytotoxins share homologous sequences, subtle differences in their conformational structures could have contributed to variable structure–activity relationships as the toxins act on different cell lines. On the other hand, the selectivity of cobra cytotoxin was infrequently reported, limiting the interpretation of the prospect of the toxin in anticancer drug development. The present study clearly demonstrated selectivity of NS-CTX and NK-CTX for the lung cancer cells but not for the prostate and the breast cancer cell lines. The finding, however, varied from that reported by Ebrahim et al. (2016) and Attarde and Pandit (2017) who observed selectivity of cobra cytotoxins in the breast cancer cell line MCF-7 (SI = 4.33–10). The discrepancy could be due to the fact that the cytotoxins were from different species (*N. naja* and *N. oxiana*), and that the normal breast cell line tested in the present study (184B5) was indeed the corresponding normal mammary gland epithelial cells, in contrast to the MCF10A cell line which was the immortalized, normal-like mammary gland epithelial cells (with fibrocystic disease background) used in the previous studies (Ebrahim et al., 2016; Attarde and Pandit, 2017).

**TABLE 3:** Comparison of cytotoxicity and cell-death mechanism in cancer cell lines of purified cytotoxins from *Naja* spp.

**Species**	**Venom Origin**	**Cytotoxin**	**Purification method/toxin identification method**	**Accession ID**	**Treatment duration (hours)**	**Tissue origin, cell line and IC_50_**		**SI**	**Cell-death mechanism method**	**Treatment dose/duration**	**Cell-death mechanism**	**References**
*Naja sumatrana*	Malaysia (wild)	Cytotoxin 2a	Resource S cation IEC and C_18_ Reverse-phase HPLC/ESI-nano-LCMS/MS	NSM_FTX01	72	Lung	Cancer: A549 = 0.88 μg/ml	2.17	Annexin V and PI stain flow cytometry	0.88 μg/ml, 72 h	Necrosis	Present study
							Normal: NL-20 = 1.91 μg/ml					
						Prostate	Cancer: PC-3 = 3.13 μg/ml	0.11		3.13 μg/ml, 72 h	Late apoptosis	
							Normal: RWPE-1 = 0.35 μg/ml					
						Breast	Cancer: MCF-7 = 9.10 μg/ml	0.68		9.10 μg/ml, 72 h	Late apoptosis	
							Normal: RWPE-1 = 6.21 μg/ml					
*Naja kaouthia*	Thailand	Cytotoxin 3	Resource S cation-IEC and C_18_ Reverse-phase HPLC/ESI-nano-LCMS/MS	P01446	72	Lung	Cancer: A549 = 1.22 μg/ml	2.26	Annexin V and PI stain flow cytometry	1.22 μg/ml, 72 h	Necrosis	Present study
							Normal: NL-20 = 2.76 μg/ml					
						Prostate	Cancer: PC-3 = 4.46 μg/ml	0.15		4.46 μg/ml, 72 h	Late apoptosis	
							Normal: RWPE-1 = 0.65 μg/ml					
						Breast	Cancer: MCF-7 = 12.23 μg/ml	0.23		12.23 μg/ml, 72 h	Late apoptosis	
							Normal: RWPE-1 = 2.83 μg/ml					
	N.A.	CT3Nk	Sephadex G50 IEC/N.A	N.A.	3	Lung	Cancer: A549 = 2.60 μM	–	–	–	–	Feofanov et al., 2005
						Peripheral blood	Cancer: HL60 = 2.60 μM					
*Naja atra*	N.A.	Cytotoxin 1	Sephadex G50 and CM Sepharose fast flow chromatography/N.A	N.A.	24	Breast	Cancer: MCF-7 = 3.95 μg/ml	–	–	–	–	Wu et al., 2013
						Liver	Cancer: H22 = 7.44 μg/ml	–	–	–	–	
						Bone marrow	Cancer: K562 = 5.77 μg/ml	–	–	–	–	
							Cancer: KG1a = 3.31 μg/ml	–	Annexin V and PI stain flow cytometry	4 μg/ml, 48 h	Necrosis	Liu et al., 2019
						Peripheral blood	Cancer: HL60 = 10.18 μg/ml	–		12 μg/ml, 12 h	Late apoptosis	
	China	Cardiotoxin/cytotoxin-III	Sephadex G50 and SP-Sephadex C-25 chromatography/N.A	N.A.	48	Breast	Cancer: MCF-7 = 2.00 μg/ml	–	Annexin V and PI stain flow cytometry	5 μg/ml, 24 h	Late apoptosis	Chiu et al., 2009
							Cancer: MDA-MB-231	–	Annexin V and PI stain flow cytometry	0.15 μM, 18 h	Late apoptosis	Lin et al., 2010
					12	Bone marrow	Cancer: K562 = 2.63 μg/ml	–	Annexin V and PI stain flow cytometry	3 μg/ml, 3 h	Early apoptosis	Chen et al., 2009
					48	Colon	Cancer: Colo205 = 4.00 μg/ml	–	Western blot: Bcl-2 and Bax expression	4 μg/ml, 12 h	Apoptosis	Tsai et al., 2006
					N.A	Oral cavity	Cancer: Ca9-22	–	Annexin V and PI stain flow cytometry	4 μg/ml, 24 h	Late apoptosis	Chien et al., 1994
*Naja haje*	N.A.	CT1Nh/CT2Nh	Sephadex G50 IEC/N.A	N.A.	3	Lung	Cancer: A549 = 116.00–132.00 μM	–	–	–	–	Feofanov et al., 2005
						Peripheral blood	Cancer: HL60 = 1.90–2.60 μM					
*Naja naja*	India	NN-32	CM-cellulose IEC and C18 Reverse-phase HPLC/MALDI-TOF	Multiple homologous accessions	48	Breast	Cancer: MCF-7 = 2.50 μg/ml	10	–	–	–	Attarde and Pandit, 2017
							Cancer: MDA-MB-231 = 6.70 μg/ml	3.73				
							Normal: MCF-10A = 25.00 μg/ml					
					24	Pleural effusion	Cancer: U937 = 2.00 μg/ml	–	Annexin V and PI stain flow cytometry	2 μg/ml, 24 h	Late apoptosis	Das et al., 2013
*Naja nigricollis*	Nigeria	Cytotoxin-(1/2/4)N	C18, C4 and C8 Reverse-phase HPLC/CID-MS/MS mass spectrometric analysis	P01452 P01467 P25517	24	Lung	Cancer: A549 = 0.80–9.00 μM	–	–	–	–	Conlon et al., 2020
						Breast	Cancer: MDA-MB-231 = 6 to > 30 μM					
						Colon	Cancer: HT-29 = 8 to >30 μM					
*Naja oxiana*	N.A.	Cytotoxin-II	CM-cellulose and SE-Sephadex C-25 IEC/N.A	N.A.	24	Breast	Cancer: MCF-7 = 4.18 μg/ml	4.33	-	-	-	Ebrahim et al., 2014
							Normal: MCF10A = 18.12 μg/ml					
	N.A.	CT1No/CT2No	Sephadex G50 IEC/N.A	N.A.	3	Lung	Cancer: A549 = 1.7–16.60 μM	–	–	–	–	Feofanov et al., 2005
						Peripheral blood	Cancer: HL60 = 0.33–0.58 μM					

*HPLC, high performance liquid chromatography; IEC, ion-exchange chromatography; IC_50_, median inhibitory concentration; N.A., not available; PI, propidium iodide; SI, selectivity index.*

In drug discovery, the cell death mechanism underlying the anticancer activity of cytotoxin is as important as its selectivity for cancer cells. Apoptosis is, in general, preferred over necrosis as necrosis-induced inflammation can be hazardous to normal tissues. The mechanistic study of cancer cell death induced by cobra cytotoxins has revolved mainly around *N. atra* and, to a small extent, *N naja* but virtually never been investigated for cytotoxins from other cobra species. The majority of cancer cell lines (breast, colon, oral cavity, and pleural effusion) treated with *N. atra* and *N. naja* cytotoxins appeared in the late apoptotic stage (Table 3), consistent with the present finding of late apoptosis in MCF-7 cell line treated with NS-CTX and NK-CTX. Furthermore, in the A549 lung cancer cell line, we observed necrosis in the cells upon treatment with NS-CTX and NK-CTX, a finding similar to that reported by Liu et al. (2019) in KG1a leukemic cell line treated with *N. atra* cytotoxin 1. The cytotoxin necrotizing pathway probably lacks attraction for further elucidation, although the authors suggested that *N. atra* cytotoxin 1 initiated necroptosis in KG1a cells that may have involved the stimulation of interferon, tumor necrosis factor, and T cell receptors (Liu et al., 2019).

## Conclusion

The development of peptide-based anticancer drug has been gaining momentum in recent years due to the possibility of improved potency, efficacy, and minimal systemic toxicity. The cobra cytotoxins have such potentials but their highly evolved, diet-adapted cytotoxic nature might have limited the intended application as a safe and effective drug. The present study demonstrated such challenges and dilemma in developing these cytotoxic proteins as anticancer therapies. The cytotoxins from *N. sumatrana* (NS-CTX) and *N. kaouthia* (NK-CTX) were highly potent in inhibiting the growth of the lung (A549), prostate (PC-3), and breast (MCF-7) cancer cell lines but lack selectivity for the latter two cell lines. Although NS-CTX and NK-CTX exhibited good selectivity in the lung A549 cancer cell line (sparing the normal NL20 cell line), the cell death pathway was predominantly by necrosis, questioning its use as a safe anticancer candidate. The study underscores the need for a comprehensive, fundamental investigation that addresses the selectivity and cell death mechanism of venom proteins, in the quest of novel anticancer agents. The anticancer potential of the toxins, however, may be further improved through structural modification to deliver a more specific, molecularly targeted cancer therapy in the future.

## Data Availability Statement

All datasets presented in this study are included in the article/Supplementary Material.

## Author Contributions

CHT and KYT conceptualized the study, designed the study and methodology, and provided resources. HPC was involved in the investigation, analysis, data validation, and writing the original draft of manuscript. All authors reviewed, edited, and approved the manuscript.

## Conflict of Interest

The authors declare that the research was conducted in the absence of any commercial or financial relationships that could be construed as a potential conflict of interest.

## Footnotes

1 https://www.ncbi.nlm.nih.gov/Taxonomy/Browser/wwwtax.cgi?mode=Info&id=8570

2 http://www.uniprot.org/

3 http://proteomecentral.proteomexchange.org
