# Supplementary material for: Cytotoxicity of Snake Venoms and Cytotoxins From Two Southeast Asian Cobras (Naja sumatrana, Naja kaouthia): Exploration of Anticancer Potential, Selectivity, and Cell Death Mechanism
Source: Front Mol Biosci. 2020 Nov 11;7:583587. doi: 10.3389/fmolb.2020.583587 (PMC7686564; doi:10.3389/fmolb.2020.583587)
Supplement: Supplementary file 1 [file Data_Sheet_1.PDF]

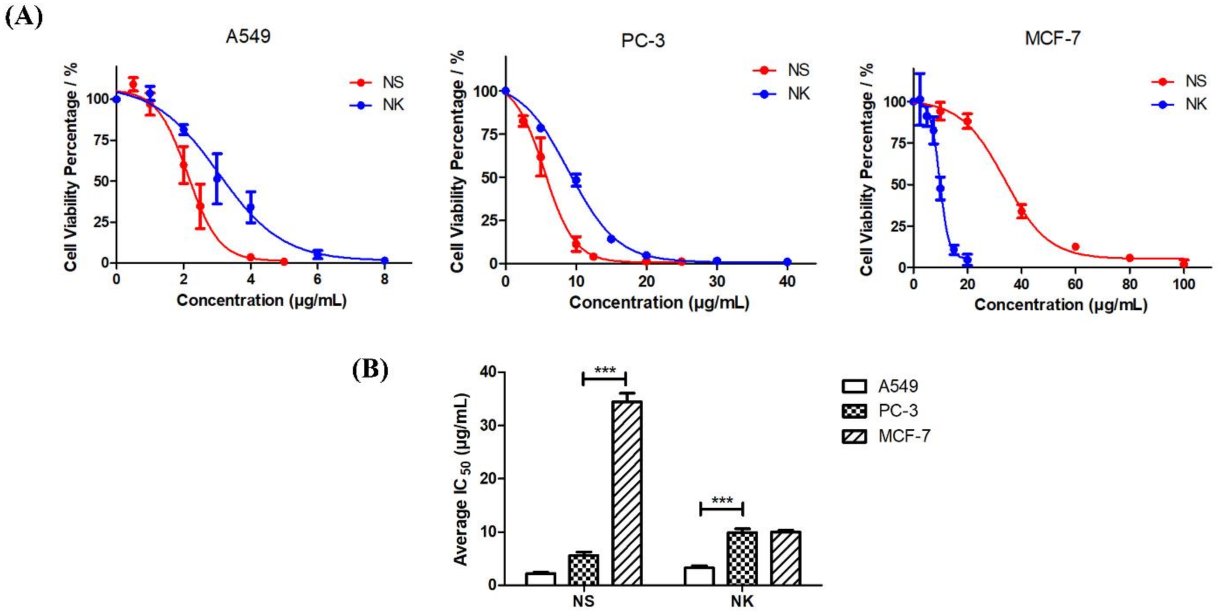

Supplementary File 1. Cytotoxicity of *Naja sumatrana* (NS) and *Naja kaouthia* (NK) venoms in A549, PC-3 and MCF-7 cancer cell lines. (A) Cell viability plot of different cell lines treated with NS and NK venoms. (B) Half maximal inhibitory concentrations (IC_{50}) of NS and NK venoms in different cell lines. All assays utilized three technical and biological triplicates. One-way ANOVA, with Bonferroni post hoc-test was utilized in determining statistical significance across three cancer cell lines. (*** indicates $p < 0.001$).
