# Supplementary material for: Cytotoxicity of Snake Venoms and Cytotoxins From Two Southeast Asian Cobras (Naja sumatrana, Naja kaouthia): Exploration of Anticancer Potential, Selectivity, and Cell Death Mechanism
Source: Front Mol Biosci. 2020 Nov 11;7:583587. doi: 10.3389/fmolb.2020.583587 (PMC7686564; doi:10.3389/fmolb.2020.583587)
Supplement: Supplementary file 2 [file Data_Sheet_2.docx]

| **Supplementary File 2. Half maximal inhibitory concentrations (IC_50_) of *N. sumatrana* and *N. kaouthia* venoms in different cancer cell lines.** | | | |
| --- | --- | --- | --- |
| **Tissue origin** | **Cell lines** | **NS (µg/ml)** | **NK (µg/ml)** |
| Lung | A549 | 2.21 ± 0.25 | 3.26 ± 0.39 |
| Prostate | PC-3 | 5.58 ± 0.67 | 9.88 ± 0.71 |
| Breast | MCF-7 | 34.47 ± 1.59 | 10.00 ± 0.30 |
